# miR-765 inhibits the osteogenic differentiation of human bone marrow mesenchymal stem cells by targeting BMP6 via regulating the BMP6/Smad1/5/9 signaling pathway

**DOI:** 10.1186/s13287-020-1579-0

**Published:** 2020-02-14

**Authors:** Tao Wang, Chao Zhang, Cihu Wu, Jianyun Liu, Hui Yu, Xiaoou Zhou, Jie Zhang, Xinping Wang, Shan He, Xiaoyuan Xu, Baicheng Ma, Xiangxin Che, Weidong Li

**Affiliations:** 1 Key Laboratory of System Bio-medicine of Jiangxi Province, Jiujiang University, Jiujiang, 332000 China; 2 Breast surgery, Affiliated Hospital of Jiujiang University, Jiujiang, 332000 China; 3 Medical department, Jiujiang University, Jiujiang, 332000 China

**Keywords:** miR-765, Human bone marrow mesenchymal stem cells, Osteogenic differentiation, Bone morphogenetic protein 6

## Abstract

**Background:**

The process of bone repair is heavily dependent on the ability of human bone marrow mesenchymal stem cells (hMSCs) to undergo osteogenic differentiation. MicroRNAs have been shown to regulate this osteogenic process. This study aimed to investigate the role of miR-765 in the osteogenic differentiation of hMSCs.

**Methods:**

We transfected hMSCs with lentiviral constructs to knock down or overexpress this miRNA, allowing us to assess its role in osteogenesis via assessing the expression of the relevant markers alkaline phosphatase (ALP), runt-related gene-2 (RUNX-2), and osteocalcin (OCN), with further functional measurements made via quantifying ALP activity and conducting Alizarin Red S staining. The targeting of the 3′-untranslated region (UTR) of BMP6 by miR-765 was examined via luciferase assay. We used hMSCs with altered miR-765 expression to assess p-Smad1/5/9 levels via Western blotting over the course of osteogenic differentiation. We also assessed the osteogenic differentiation of hMSCs in which miR-765 was knocked down and at the same time as a BMP/Smad signaling inhibitor was added to disrupt Smad1/5/9 phosphorylation.

**Results:**

We found miR-765 overexpression to inhibit osteogenesis-associated gene upregulation during osteogenic differentiation of hMSCs, whereas knockdown of this miRNA was associated with increased expression of these genes. Using luciferase reporter assays, we confirmed direct miR-765 binding to the 3′-untranslated region (UTR) of BMP6. We also found that miR-765 overexpression reduced Smad1/5/9 phosphorylation, and knockdown of this miRNA enhanced this phosphorylation on BMP6/Smad1/5/9 signaling. The osteogenic differentiation of hMSCs in which miR-765 had been knocked down was further weakened upon the addition of a BMP/Smad signaling inhibitor relative to miR-765 knockdown alone.

**Conclusions:**

Together, these results thus suggest that miR-765 is able to inhibit hMSC osteogenic differentiation by targeting BMP6 via regulation of the BMP6/Smad1/5/9 signaling pathway. Our findings may offer molecular insights of value for the development of novel therapeutic treatments for bone diseases including osteoporosis.

## Background

Human mesenchymal stem cells (hMSCs) are self-renewing cells that can be isolated from human bone marrow and which can differentiate into several different cell types [1]. In vivo, these cells can undergo osteoblastic or adipocytic differentiation in response to appropriate stimuli [2–5]. Disruption of the normal homeostasis between osteoblast and adipocyte differentiation such that relative osteoblast levels are reduced can result in an overall decrease in bone mass as is characteristic of osteoporosis [6]. The specific mechanisms controlling hMSC differentiation have thus been an area of active research, with in vitro expanded hMSCs representing an ideal model system for identifying the regulators of this process [7].

MicroRNAs (miRNAs) are small non-coding RNAs that are able to bind the 3′-untranslated region (UTR) of specific complementary mRNAs, thereby promoting their degradation or translational repression [8]. Several miRNAs have been shown to be linked to osteogenesis, including miR-206, miR-204, and miR-214, all of which can inhibit this process [9–11]. In contrast, expression of miR-15b and miR-29b has been found to be pro-osteogenic [12, 13]. This suggests that differential miRNA expression is a key mechanism regulating osteogenic differentiation. The specific regulatory role of miR-765, which is encoded on Chromosome 1 (NC_000001.11; 156936131...156936244) in osteogenesis, however, remains uncertain.

Bone morphogenetic protein (BMP) is a protein that is essential for the normal repair of fractures [14]. Osteoblasts synthesize and secrete these BMP proteins, releasing them in large quantities into the bone matrix environment [15]. In total, there are over 20 BMP family proteins, and they are known to be able to strongly promote osteogenesis via direct stimulation of hMSCs leading to their differentiation [16, 17]. Recent studies have explored the role of miRNAs in the regulation of BMPs within osteoblasts [18–21]. While BMP6 has been shown to play a key role in the osteogenic process, as has miR-765 which binds to the BMP6 mRNA, the specific underlying signaling mechanisms remain unclear.

In the present study, we assessed the importance of miR-765 in hMSC osteoblastic differentiation, revealing this miRNA to control osteogenesis by targeting BMP6 via regulation of the BMP6/Smad1/5/9 signaling pathway.

## Materials

### Cell culture and osteogenic differentiation

hMSCs (HUXMA-01001, Cyagen Biosciences, China; cell lot no. of three donors: 150724I31, 160202I31, and 161125R41) were phenotyped via flow cytometry and were confirmed to be ≥ 95% CD73-, CD90-, and CD105-positive, as well as CD11b-, CD19-, CD45-, CD34-, and CD HLA-DR-negative (≤ 5%) (Additional file 1). These cells were initially plated at 5 × 10^4^ cells/cm^2^ in oriCellTM human Mesenchymal Stem Cell growth media (HUXMA-90011, Cyagen Biosciences) supplemented with 10% FBS, glutamine, and penicillin/streptomycin in a 5% CO_2_ humidified incubator at 37 °C with 95% air. Every 3–4 days, 0.25% trypsin-EDTA (Gibco; Thermo Fisher Scientific, Inc.) was used to detach cells, which were then passaged. All experiments were conducted with cells from passages 3–6.

To induce osteogenic differentiation, cells were allowed to grow until 70% confluent at which time they were treated with 50 mM ascorbic acid, 10 mM β-glycerophosphate, and 100 nM dexamethasone (all from Sigma-Aldrich, St. Louis, MO) for 20 days, with media being changed every 3 days.

### Lentiviral transfection

In order to overexpress or knock down miR-765, appropriate lentiviral vectors were obtained from Shanghai Genechem Co., Ltd. A short hairpin RNA (shRNA) targeting human miR-765 was designed (target sequence, 5′-CATCACCTTCCTTCTCCTCCA-3′). In addition, a negative control (NC) shRNA was used (sequence, 5′-TTCTCCGAACGTGTCACGT-3′). Chemically synthesized DNA oligonucleotides (Shanghai Genechem Co., Ltd.) were respectively cloned into the pGV280 (for shRNA) and pGV369 (for overexpression)-green fluorescent protein (GFP) vector lentiviruses (Shanghai Genechem Co., Ltd.). Cells were transfected to either overexpress miR-765, overexpress a control construct, express a miR-765-specific shRNA, or express a control shRNA. Lentiviral titers were assessed via serial dilution, and hMSCs were added to 6-well plates at 5 × 10^4^ cells/cm^2^ and allowed to grow until 20–30% confluent. At that time, 1 × 10^8^ TU/ml lentivirus (in 10 μl) was added to cells along with 5 μg/ml polybrene and complete medium. Cells were then allowed to incubate for 10 h at 37 °C, after which fresh medium was added and cells were grown for 72 h. After that time, antibiotic selection was conducted by adding 0.5 μg/ml puromycin to cells, replacing medium every 1–2 days for 6 days in total until surviving cells appeared to proliferate readily.

### Alizarin Red S staining

Cells were washed thrice using PBS and were then fixed for 20 min with cold ethanol (95%) and air dried. Next, 0.5% ammonium hydroxide (Sigma-Aldrich, MO) was generated through appropriate dilution of a 30% stock solution in dH_2_O. Then, Alizarin Red S (ARS) (Sigma-Aldrich, MO) was dissolved in dH_2_O at a 40-mM concentration, and the ammonium hydroxide solution was used to achieve a pH between 4.1 and 4.3. This ARS solution was then used to stain samples for 30 min at room temperature, followed by three washes using dH_2_O. Next, 10% (w/v) cetylpyridinium chloride (Sigma-Aldrich, MO) was prepared in 0.1 M sodium phosphate buffer (pH 7.0), and this was used for destaining. Samples were then analyzed based on their absorbance at 560 nm.

### Assessment of ALP activity

A commercial ALP Detection Kit (Nanjing Jiancheng Bioengineering Ltd., Nanjing, China) was utilized based on provided protocols. Cells were first frozen and thawed to room temperature four times in order to mediate ALP release. Cell lysates were then added to 96-well plates and combined for 30 min with the ALP substrate at 37 °C, after which a stop buffer was added. Sample absorbance at 560 nm was then assessed via microplate reader (Biorad, Hercules, CA) to measure the enzymatic hydrolysis of the p-nitrophenyl phosphate substrate.

### Quantitative real-time PCR

TRIzol (Invitrogen) was used for total RNA extraction based on provided protocols. A Reverse Transcription System and Oligo (dT) was used to generate cDNA based on provided directions (Thermo Scientific). A stem-loop real-time PCR miRNA kit (Ribobio, China) was used for quantifying miR-765 expression, with U6 as an internal control using primers from Ribobio. For mRNA measurements, β-actin served as a normalization control. Primers used herein are shown in Table 1. A SYBR Premix Ex Taq kit (TOYOBO, Japan) was used for conducting qRT-PCR reactions with a 7500 Real-Time PCR System (ABI, Foster, CA, USA), and the 2^−ΔΔCT^ method was used for quantifying gene expression.

**Table 1:** Primer sequences

Gene symbol	Forward primers	Reverse primers	Length (bp)
RUNX2	5′-GGACGAGGCAAGAGTTTCACC-3′	5′-GGTTCCCGAGGTCCATCTACT-3′	161
OCN	5′-TGAGAGCCCTCACACTCCTC-3′	5′-CGCCTGGGTCTCTTCACTAC-3′	151
ALP	5′-CCCCGTGGCAACTCTATCTTT-3′	5′-GCCTGGTAGTTGTTGTGAGCATAG-3′	161
BMP6	5′-ACAGCATAACATGGGGCTTC-3′	5′-GAAGGGCTGCTTGTCGTAAG-3′	112
β-actin	5′-GCGAGAAGATGACCCAGATCATGT-3′	5′-TACCCCTCGTAGATGGGCACA-3′	160

### Western blotting

Cells were lysed using RIPA buffer (50 mM Tris (pH 7.4), 150 mM NaCl, 1% Triton X-100, 0.1% SDS, 1 mmol/l sodium orthovanadate, 1% sodium deoxycholate, 1 mM EDTA, 2 μg/ml leupeptin, 50 mmol/l sodium fluoride). Protein samples were boiled in 5× SDS sample buffer for 5 min, and 15 μg of each samples was separated via 10% SDS-PAGE and transferred onto PVDF membranes (EMD Millipore). Next, 5% skim milk was used to block membranes for 120 min at room temperature, and blots were then probed at 4 °C overnight using the following antibodies: rabbit anti-RUNX2 (ab23981), rabbit anti-OCN (ab133612), rabbit anti-BMP6 (AF5196), rabbit anti-Phospho-Smad1/5/9 (AF8313), rabbit anti-Smad1/5/9 (AF0614), and mouse anti-β-actin (1:2000; ab173838). All rabbit antibodies were diluted 1:1000. Blots were then probed at room temperature for 60 min with an HRP-conjugated secondary antibody specific for either mouse IgG or rabbit IgG (1:5000; Cell Signaling Technology). Protein was then visualized via enhanced chemiluminescence (BeyoECL Plus; Beyotime Institute of Biotechnology).

### miRNA target gene prediction

The TargetScan 6.2, PicTar, and miRBase 21 algorithms were used as a means of identifying possible miR-765 target genes.

### Luciferase reporter assay

A Dual-Luciferase Reporter Assay System (pGL3 vector; Promega, USA) was used to validate BMP6 as a miR-765 target gene. The BMP6 3′-UTR region containing the putative miRNA binding site of interest was added to the pGL3 vector downstream of luciferase, resulting in the production of either a pGL3-wild-type (WT) or a pGL3-mutant (MUT) vector, with DNA sequencing used to confirm vector construction. These vectors were then transfected into 293T cells either with or without a miR-765 mimic. After 48 h, luciferase activity was assessed based on provided instructions, with Renilla luminescence used to normalize luciferase activity relative to miRNA-transfected controls.

### Statistical analysis

Data are means ± standard deviation of experiments repeated three times. SPSS (v16.0; SPSS, Inc.) was used to compare data via one-way ANOVAs, with *P* < 0.05 as the significance threshold.

## Results

### miR-765 expression decreases during osteogenic differentiation

**Fig. 1:**
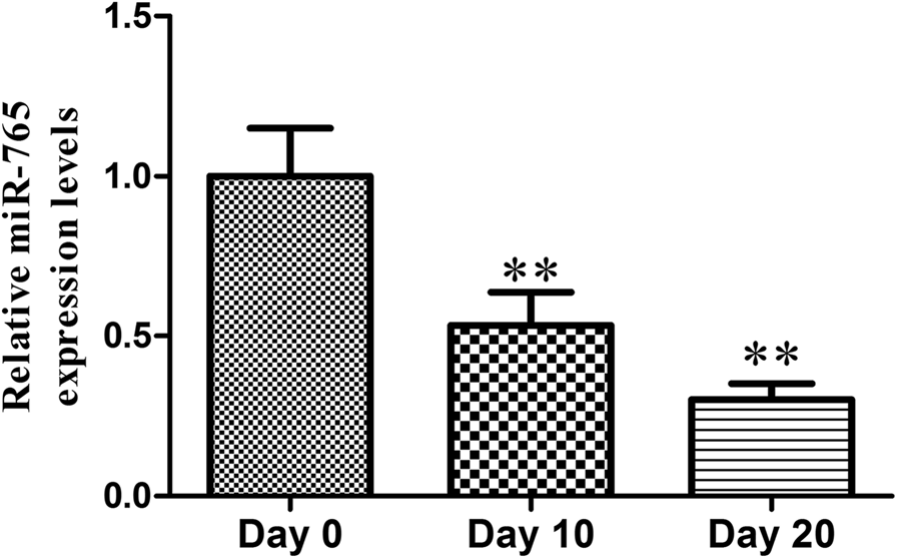
miR-765 expression over the course of osteogenic differentiation was assessed via qRT-PCR at indicated time points. All values are expressed as means ± SD (*X* ± SD, *n* = 3). **P < 0.01 vs. day 0

We first found that miR-765 expression gradually decreases over the course of osteogenesis, indicating that it may play a key role in regulating hMSC osteogenic differentiation (Fig. 1).

### Generation of hMSCs with altered miR-765 expression

**Fig. 2:**
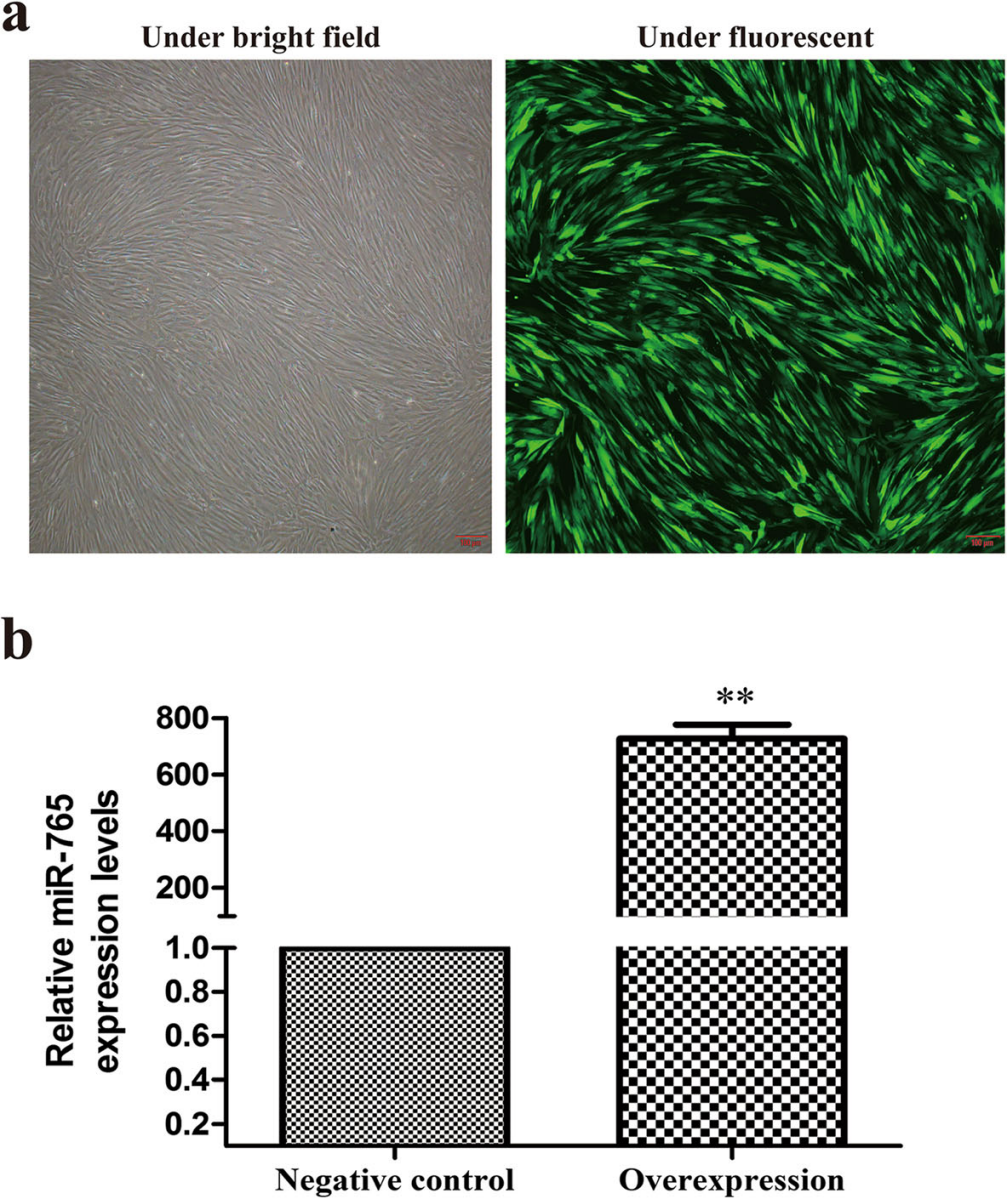
Stably infected hMSC identification. **a** Infected cells were assessed via both light and fluorescent microscopy(4×); scale bar, 100 μm. A single representative image is shown. **b** miR-765 expression was assessed via qRT-PCR. All values are expressed as means ± SD (*X* ± SD, *n* = 3). **P < 0.01 vs. negative control

We next generated cells stably expressing vectors that led them to either overexpress miR-765 or an shRNA targeting this miRNA, or to express control vectors. Following puromycin selection, we observed good growth and GFP fluorescence from surviving cells consistent with successful stable transfection (Fig. 2a). We then verified that the lentiviral transfection was successful via qRT-PCR measurement of miR-765, revealing the cells overexpressing miR-765 to have a > 700-fold increase in the expression of this miRNA relative to negative control cells (Fig. 2b).

### miR-765 inhibits the osteogenic differentiation of hMSCs

**Fig. 3:**
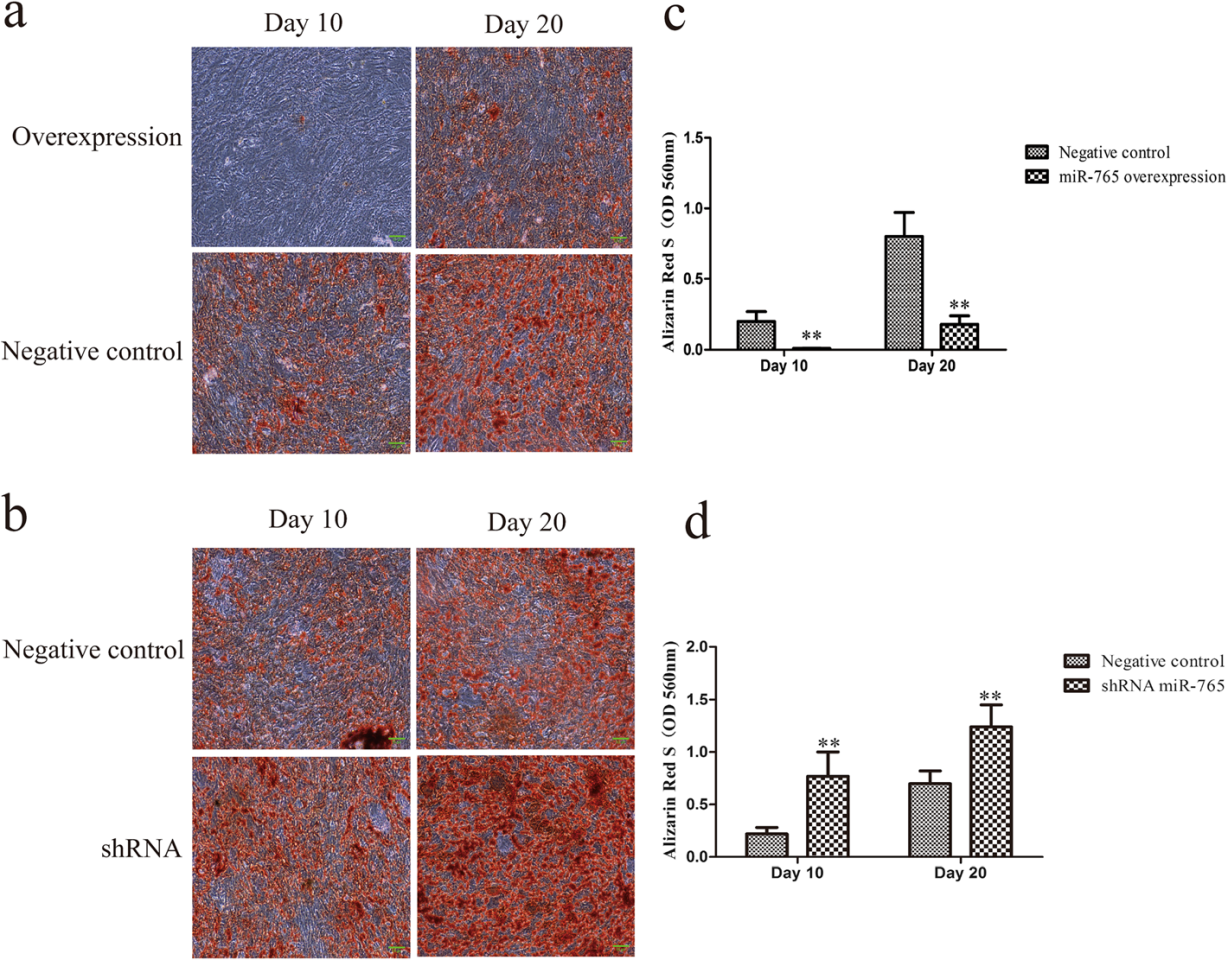
miR-765 inhibits hMSC osteogenic differentiation. Alizarin Red S staining was employed as a means of assessing matrix mineralization. Scale bar, 50 μm. **a** miR-765 overexpression was associated with decreased matrix mineralization. **b** Cell in which miR-765 had been knocked down exhibited more intense staining consistent with increased mineralization. **c**, **d** Differences in mineralization staining intensity were significant among these groups of cells. Data are expressed as means ± SD (*X* ± SD, *n* = 3). **P < 0.01 vs. negative controls, respectively

To explore how miR-765 influences osteogenic differentiation, we assessed osteogenesis in hMSCs in which miR-765 was knocked down or overexpressed on days 0, 10, and 20 following the addition of osteogenic medium. When we conducted Alizarin Red S staining on days 10 and 20, we found that miR-765 overexpression was associated with reduced staining intensity relative to control cells, suggesting decreased matrix mineralization (Fig. 3a). In contrast, cells in which miR-765 had been knocked down exhibited more intense staining consistent with increased mineralization (Fig. 3b). Differences in mineralization staining intensity were significant among these groups of cells at both days 10 and 20 (Fig. 3c, d), suggesting that miR-765 plays a key role in regulating the osteogenesis of hMSCs.

**Fig. 4:**
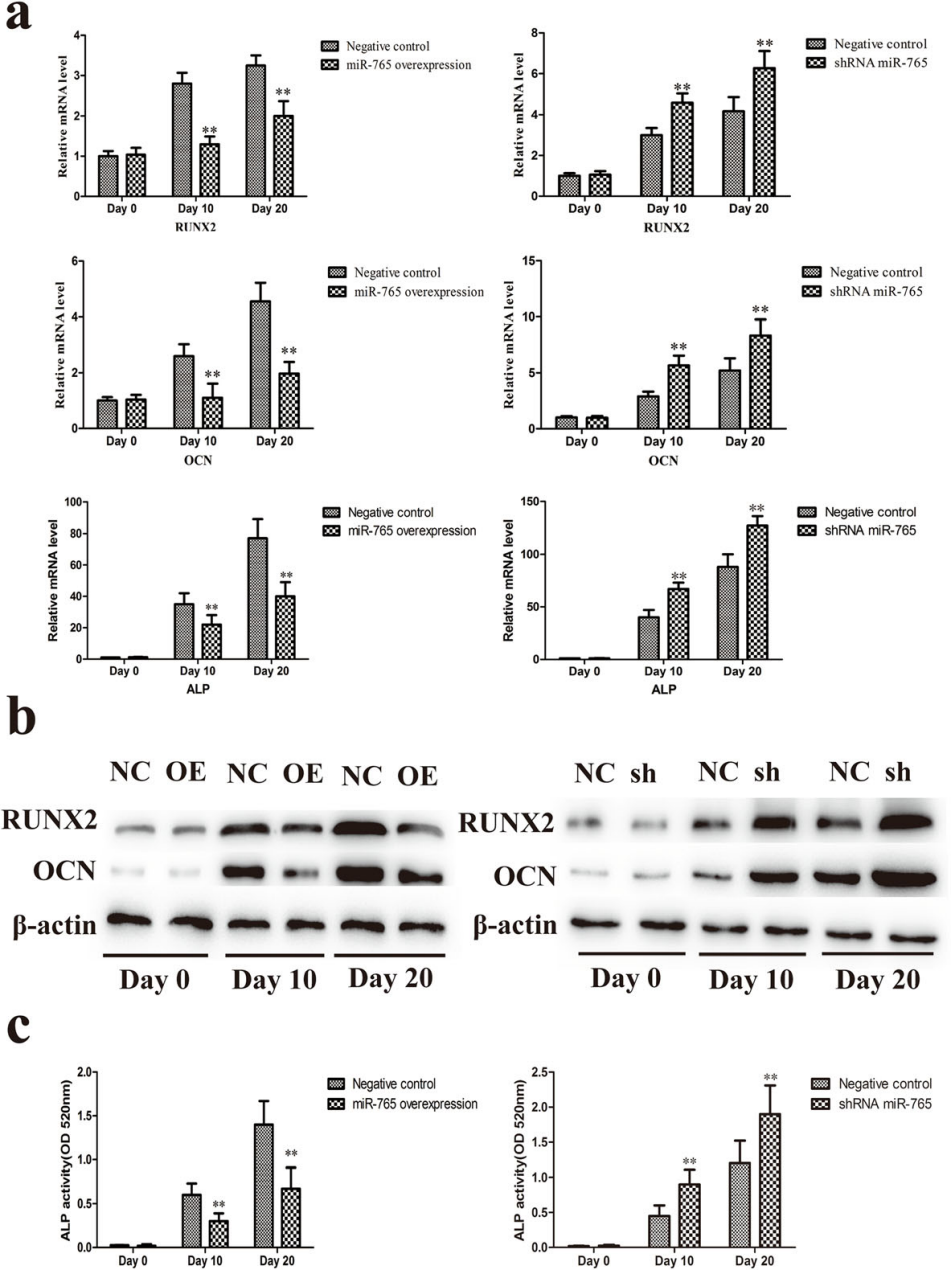
miR-765 inhibits hMSC osteogenic differentiation. **a** qRT-PCR was used to compare RUNX2, OCN, and ALP expression in the different groups. **b** RUNX2 and OCN protein levels were assessed via Western blotting. **c** ALP activity was quantified. Data are expressed as means ± SD (*X* ± SD, *n* = 3). **P < 0.01 vs. negative controls, respectively. Note: NC: negative control; OE: miR-765 overexpression; sh: shRNA

To further explore this process, we next measured expression of the osteogenic markers RUNX2 and OCN via qRT-PCR and Western blotting. We found that miR-765 overexpression was associated with a significant repression of RUNX2 and OCN expression, whereas knockdown of this miRNA was associated with increased expression of both of these genes (Fig. 4a, b). We additionally found miR-765 overexpression to impair ALP activity, whereas this activity was enhanced in cells in which this miRNA had been knocked down (Fig. 4c). This suggests that miR-765 plays a key role in hMSC osteogenesis.

### miR-765 and BMP6 expression are negatively correlated with one another during osteogenic differentiation

**Fig. 5:**
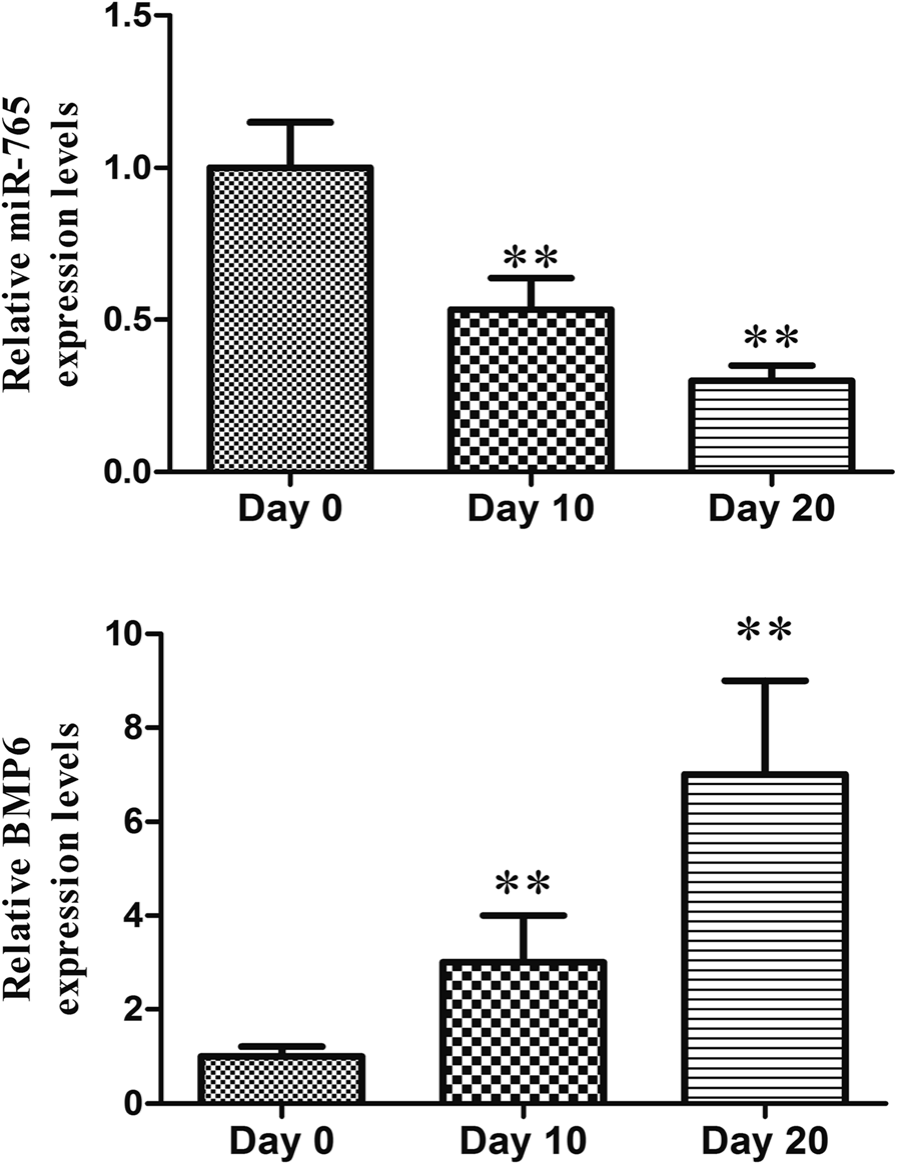
miR-765 and BMP6 expression over the course of osteogenic differentiation. BMP6 and miR-765 expression were assessed via qRT-PCR in individual groups. Data are expressed as means ± SD (*X* ± SD, *n* = 3). **P < 0.01 vs. day 0

BMP6 as a putative target gene of miR-765, we next assessed the relative expression of these two factors in the context of osteogenesis via qRT-PCR. We found that there was a clear negative correlation between the expression of miR-765 and BMP6 during hMSC osteogenic differentiation (Fig. 5).

### BMP6 is a direct target of miR-765

**Fig. 6:**
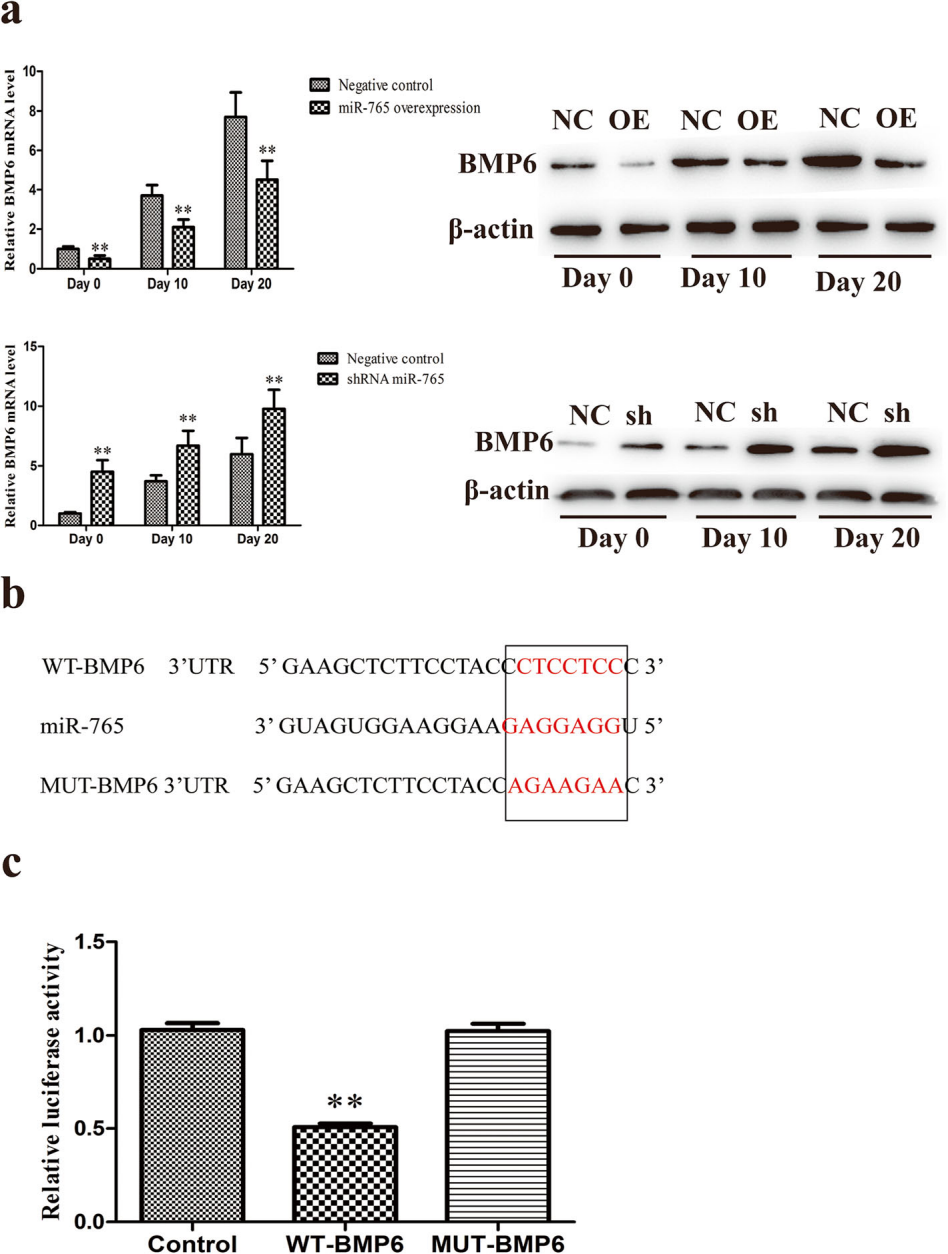
miR-765 targets the 3′-UTR of BMP6. **a** The ability of miR-765 to impact BMP6 expression over the course of osteogenic differentiation in hMSCs following stable lentiviral transfected was assessed. **b** The miR-765 binding region in the BMP6 3′-UTR. **c** Luciferase activity assay. All values are expressed as means ± SD (*X* ± SD, *n* = 3). **P < 0.01 vs. negative controls, respectively. Note: NC: negative control; OE: miR-765 overexpression; sh: shRNA

We next sought to directly assess the ability of miR-765 to regulate hMSC osteogenic differentiation via targeting BMP6. We therefore assessed the impact of miR-765 on BMP6 expression at different stages of the osteogenic differentiation process (Fig. 6a). Based on TargetScan analyses, we identified a miR-765 targeting site located within the BMP6 3′-UTR (Fig. 6b). To confirm that this was indeed a binding site for this miRNA, we utilized a standard luciferase reporter assay. This assay confirmed that there was a 50% reduction in luciferase activity in cells expressing the WT version of this sequence that had been transfected with miR-765 relative to those that had not. In contrast, cells expressing the mutated version of this construct did not exhibit any reduction in luciferase activity upon miR-765 transfection (Fig. 6c).

### miR-765 derepresses BMP6/Smad1/5/9 signaling during osteogenic differentiation

To assess whether miR-765 expression has any influence on Smad signaling in the context of osteogenesis, we used our stable hMSC constructs to assess p-Smad1/5/9 levels by Western blotting over the course of osteogenic differentiation. We found that miR-765 overexpression was associated with reduced p-Smad1/5/9 levels, whereas miR-765 knockdown was associated with increased p-Smad1/5/9 levels relative to appropriate controls (Fig. 9a, b).

**Fig. 7:**
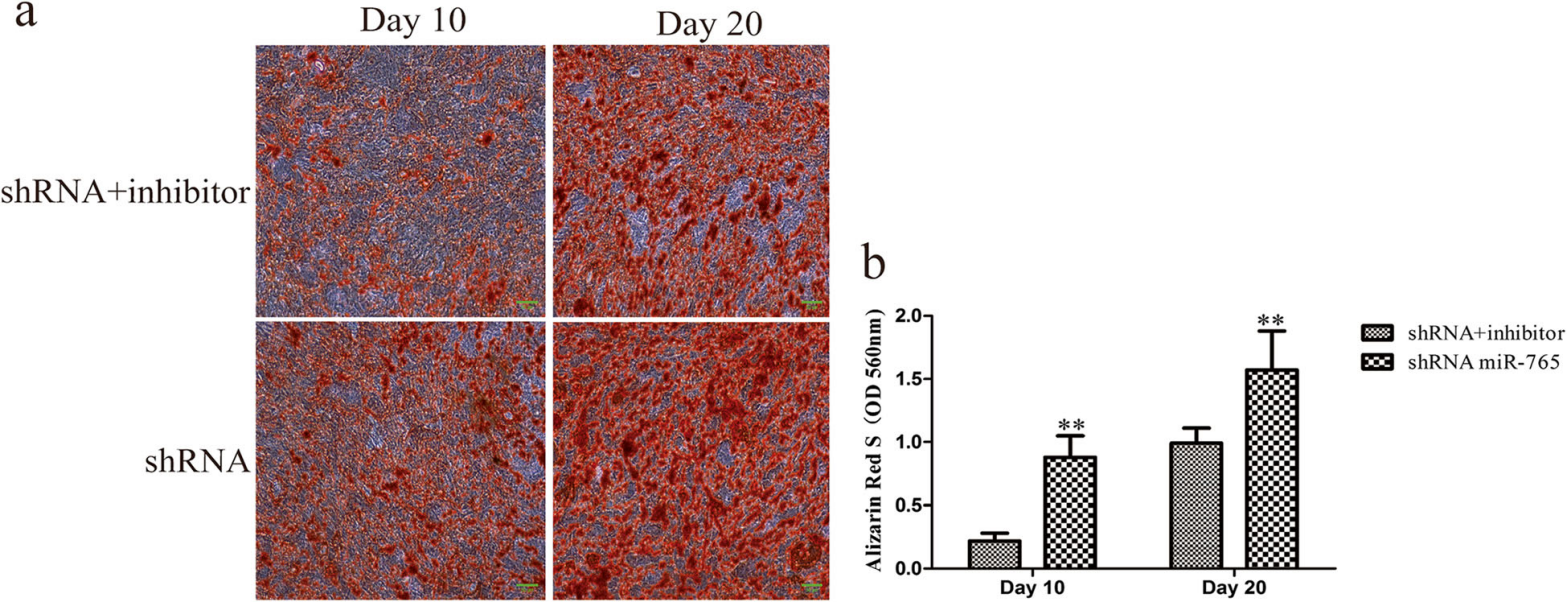
The osteogenic differentiation of hMSCs in which miR-765 was knocked down and at the same time as a BMP/Smad signaling inhibitor was added to disrupt Smad1/5/9 phosphorylation. Alizarin Red S staining was employed as a means of assessing matrix mineralization. Scale bar, 50 μm. **a** By adding a BMP/Smad signaling inhibitor, the osteogenic differentiation of hMSCs in which miR-765 was also knocked down was associated with decreased matrix mineralization relative to cells in which miR-765 was knocked down but no inhibitor was added. **b** Differences in mineralization staining intensity were significant between shRNA+inhibitor and shRNA groups. Data are expressed as means ± SD (*X* ± SD, *n* = 3). **P < 0.01 vs. shRNA+inhibitor, respectively

**Fig. 8:**
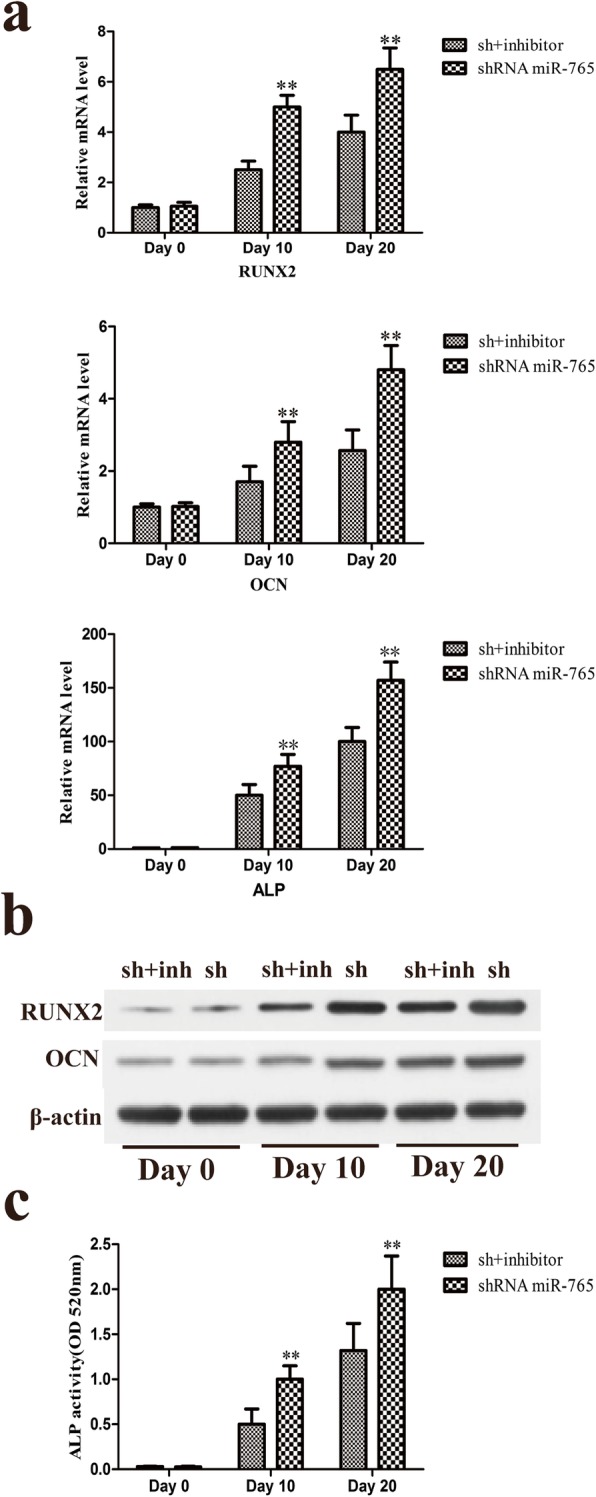
The osteogenic differentiation of hMSCs in which miR-765 was knocked down and at the same time as a BMP/Smad signaling inhibitor was added to block Smad1/5/9 phosphorylation. **a** qRT-PCR was used to compare RUNX2, OCN, and ALP expression in the different groups. **b** RUNX2 and OCN protein levels were assessed via Western blotting. **c** ALP activity was quantified. Data are expressed as means ± SD (*X* ± SD, *n* = 3). **P < 0.01 vs. shRNA+inhibitor, respectively. Note: sh: shRNA; sh+inh: sh+inhibitor

**Fig. 9:**
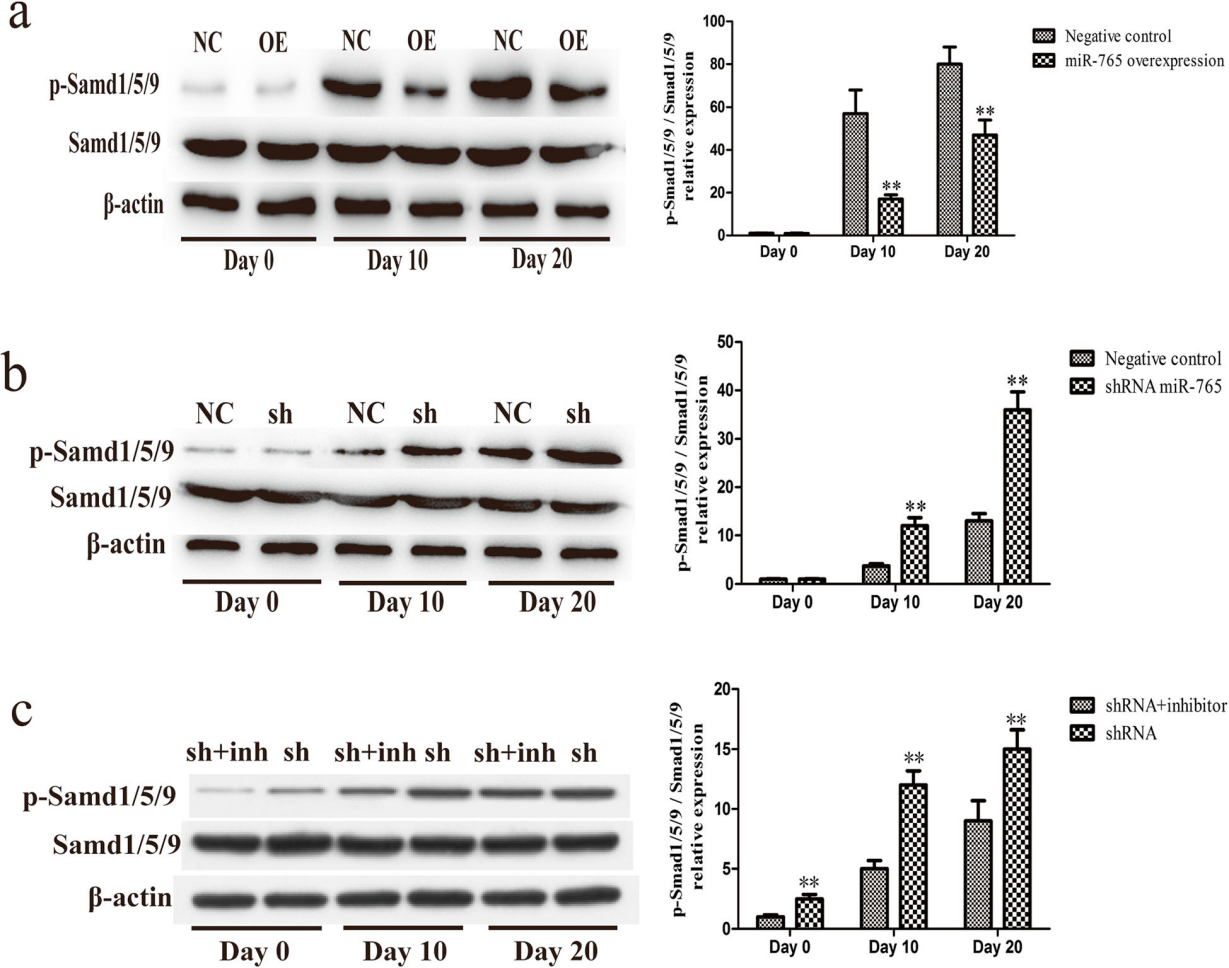
miR-765 suppresses BMP6/Smad1/5/9 signaling to inhibit osteogenesis. Western blotting was used to measure p-Smad1/5/9 levels, with β-actin as a loading control. **a** miR-765 overexpression was associated with reduced Smad1/5/9 phosphorylation upon osteogenic differentiation. **b** miR-765 knockdown significantly enhanced p-Smad1/5/9 levels. **c** By adding a BMP/Smad signaling inhibitor to reduced p-Smad1/5/9 levels. BMP6/Smad1/5/9 was shown to be downstream of miR-765, with miR-765 controlling the activation of this pathway. Data are expressed as means ± SD (*X* ± SD, *n* = 3). **P < 0.01 vs. negative controls or shRNA+inhibitor, respectively. Note: NC: negative control; OE: miR-765 overexpression; sh: shRNA; sh+inh: sh+inhibitor

Cells in which miR-765 had been knocked down were treated with 100 nM LDN-193189 (Selleck, USA), a small molecule inhibitor of BMP type I receptor that was used to specifically inhibit BMP/Smad signaling, in order to reduce p-Smad1/5/9 levels in these cells (Fig. 9c), revealing that hMSC osteogenic differentiation in cells in which miR-765 had been knocked down was weaker as compared with that in cells in which miR-765 had been knocked down in the absence of inhibitor addition (Figs. 7, 8). These results thus further revealed that BMP6/Smad1/5/9 is downstream of miR-765.

## Discussion

Recent work has clearly highlighted the role of endogenous miRNAs as key regulators of stem cell differentiation and renewal [22, 23]. Consistent with this, in the present study, we observed the gradual downregulation of miR-765 in hMSCs as they underwent osteogenic differentiation (Fig. 1). This suggested that this miRNA may play a key role in this osteogenic process, leading us to generate hMSCs stably overexpressing miR-765 or an shRNA targeting this miRNA (Fig. 2a, b). When we used these cells to assess changes in osteogenesis, we found miR-765 overexpression to significantly impair ALP activity and matrix mineralization, whereas knockdown of this miRNA had the opposite effect (Fig. 3 and Fig. 4c). Consistent with this, miR-765 overexpression impaired the expression of osteogenic markers in hMSCs during osteogenic differentiation, while knocking down this miRNA increased the expression of these markers in this same context (Fig. 4a, b). This thus indicated that miR-765 acts to negatively regulate the osteogenic differentiation of hMSCs.

Previous work has shown that miR-765 plays a role in many contexts, including arteriosclerosis, multiple myeloma, tongue squamous carcinoma, hepatoma cell proliferation, osteosarcoma cell migration, and neural stem cell differentiation [24–29]. The role of miR-765 in hMSC osteogenic differentiation, however, has not been clearly confirmed in previous studies. Our study was able to confirm a central role for miR-765 in this process.

Developing a better understanding of how miR-765 regulates osteogenesis has the potential to highlight novel therapeutic strategies for treating osteoporosis. We therefore investigated potential miR-765 target genes, identifying and confirming BMP6 as a miR-765 target gene, the expression of which was negatively correlated with that of miR-765 (Fig. 5).

To confirm that BMP6 was truly a functional target of miR-765, we next assessed changes in BMP6 expression upon modulation of miR-765 expression. We found that miR-765 overexpression was linked to a significant drop in BMP6 expression, whereas knockdown of this miRNA markedly enhanced BMP6 expression (Fig. 6a). miRNAs inhibit target mRNA translation via binding to complementary sequences in the 3′-UTR region of these mRNAs, thereafter mediating their degradation [30]. We therefore next used a standard luciferase reporter assay to validate the presence of a direct interaction between miR-765 and the BMP6 3′-UTR, with our results confirming BMP6 to be a miR-765 target (Fig. 6b, c). This is the first study we are aware of to have demonstrated that miR-765 inhibits the osteogenic differentiation of hMSC via targeting BMP6.

BMP6 is a protein that plays key roles in the formation of bone and in the regulation of osteoporosis, bone cancer, and fracture healing [31–34]. BMP6 is a member of the transforming growth factor β (TGF-β) superfamily, which is able to bind the BMPR class II receptor and then recruits a BMPRI-type receptor ultimately leading to Smad1/5/9 protein phosphorylation and activation. Upon activation, p-Smad1/5/9 is then able to cooperate with Smad4 to translocate into the nucleus wherein it can regulate the expression of genes related to diverse processes such as differentiation, growth, proliferation, migration, and apoptosis [16, 35–37]. This thus suggested that this BMP6/Smad1/5/9 pathway is likely to be a key regulatory mechanism in the context of hMSC osteogenic differentiation. As this pathway has not been studied in this context previously, we chose to examine it in depth, revealing that miR-765 overexpression was associated with reduced Smad1/5/9 phosphorylation upon osteogenic differentiation, whereas miR-765 knockdown significantly enhanced p-Smad1/5/9 levels, suggesting that this BMP6/Smad1/5/9 signaling pathway plays a key role in osteogenic differentiation and that miR-765 controls its activation (Fig. 9a, b). Furthermore, by adding a BMP/Smad signaling inhibitor to reduce p-Smad1/5/9 levels (Fig. 9c), we were able to demonstrate that the osteogenic differentiation of hMSCs in which miR-765 was also knocked down was weaker than in cells in which miR-765 was knocked down but no inhibitor was added (Figs. 7 and 8). These results thus further confirmed that BMP6/Smad1/5/9 is downstream of miR-765, with miR-765 controlling the activation of this pathway.

## Conclusions

In summary, our results indicate that miR-765 is able to suppress hMSC osteogenic differentiation through targeting BMP6, thereby regulating the BMP6/Smad1/5/9 signaling pathway. These findings may offer novel opportunities to develop therapeutic regimens for the treatment of osteoporosis or other bone diseases.

## Supplementary information

**Additional file 1.** Dentification of hMSCs via flow cytometry.

## Data Availability

All data generated and/or analyzed during this study are included in this published article.

## Abbreviations

ALP: Alkaline phosphatase
BMP6: Bone morphogenetic protein 6
hMSCs: Human bone marrow-derived mesenchymal stem cells
miRNA: MicroRNA
OCN: Osteocalcin
qRT-PCR: Quantitative real-time polymerase chain reaction
RUNX2: Runt-related gene-2
UTR: Untranslated region
